# Microbial activity and community level physiological profiles (CLPP) of soil under the cultivation of spring rape with the Roundup 360 SL herbicide

**DOI:** 10.1007/s40201-021-00753-3

**Published:** 2021-11-12

**Authors:** Stefania Jezierska-Tys, Jolanta Joniec, Agnieszka Mocek-Płóciniak, Anna Gałązka, Joanna Bednarz, Karolina Furtak

**Affiliations:** 1 Department of Environmental Microbiology, Faculty of Agrobioengineering, University of Life Sciences in Lublin, Lublin, Poland; 2 Department of General and Environmental Microbiology, Faculty of Agronomy and Bioengineering, Poznań University of Life Sciences, Poznań, Poland; 3 Department of Agricultural Microbiology, Institute of Soil Science and Plant Cultivation, State Research Institute, Puławy, Poland

**Keywords:** Rapeseed, Biochemical and enzymatic activity, Catabolic diversity, Herbicide, Soil microorganisms

## Abstract

**Purpose:**

The use of glyphosate in agriculture raises a lot of controversy because research concerning its impact on the soil provides contradictory information. However, despite these negative opinions, glyphosate is still used in agricultural practice. Therefore, for a more complete assessment, the authors carried out research using traditional microbiological methods and a modern method of metabolic profile analysis in glyphosate-treated soil.

**Methods:**

The study was carried out on the soil witch was sown with six cultivars of rapeseed. Seven days before harvest, the plants were sprayed with the herbicide. The analyses consisted in determining the number of selected groups of microorganisms, biochemical and enzymatic activity, and differentiation of the catabolic potential of soil microbial communities.

**Results:**

The results showed significant changes in the analyzed parameters. Respiratory activity and ammonification processes were stimulated in the treatments with rapeseed cultivation treated with the herbicide. Changes in the enzymatic activity were generally positive. The EcoPlate assessment of microbial community catabolism showed that the highest activity was recorded in the soil sown with the cultivars Belinda, Tamarin, and Sw svinto. Concurrently, these soils were characterized by the highest correlations between rapeseed cultivar and metabolic activity.

**Conclusion:**

Cultivation of specific plant varieties that reduce the negative effect of herbicides used in agriculture may be one of the methods to prevent soil degradation. In our research, Belinda turned out to be a cultivar, under the cultivation of which an increase in the activity of microorganisms was recorded most frequently compared to soil not sown with rapeseed.

**Supplementary Information:**

The online version contains supplementary material available at 10.1007/s40201-021-00753-3.

## Graphical abstract

- *These studies were undertaken due to the importance of glyphosate in soil environment degradation.*
- *The results showed significant changes in the activity and diversity of microorganisms*
- *The nature and intensity of these changes depended on the time point and oilseed rape cultivar.*
- *The most favorable soil conditions for microorganisms were under the cultivar Belinda*

**Supplementary Information:**

The online version contains supplementary material available at 10.1007/s40201-021-00753-3.

## Graphical abstract

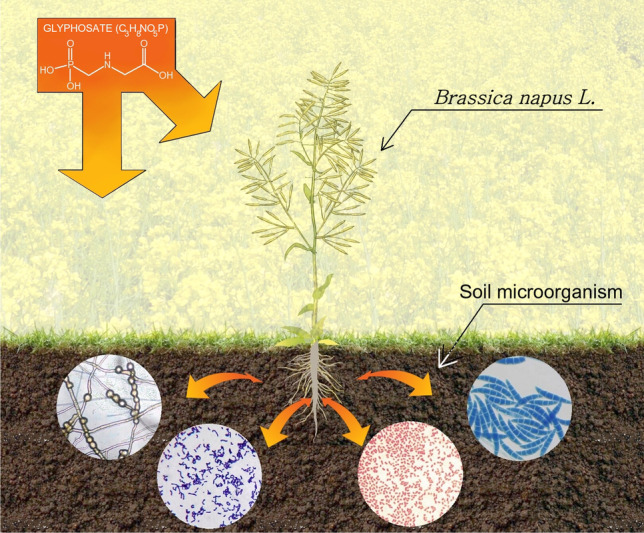

## Introduction

Herbicides are biologically active compounds that prevent growth of competitive plants in crop cultivation when used in accordance with the manufacturer’s instructions, thereby providing the desired plants with better growth and yielding conditions. The large-scale use of herbicides raises great concerns due to their contribution to contamination of the environment, which leads to soil degradation; it is associated with the risk of reducing or changing the activity of soil microorganisms [45], which can indirectly affect plant growth and other soil functions. An important role is played by crop plants, in particular their rhizosphere, which is a site of three-way interactions between the plant, roots, and microorganisms [19, 33]. Plants exert an effect on the local microclimate and soil properties as well as soil microbiological diversity. In consequence, they have a significant impact on C and N transformations and CO_2_ emission [55]. Studies show that glyphosate is biodegraded by soil microorganisms. This process requires appropriate conditions, including C abundance and adequate soil pH [28]. Bacteria and soil fungi use pesticides as a source of energy by metabolizing them in enzymatic processes [57]. Therefore, the addition of pesticides may cause an increase in enzymatic activity of the soil. The effect of glyphosate on soil microorganisms and its bioavailability and biodegradability depends on microbiome composition as well as soil properties and the type of preparation applied [6, 17].

Assessment of changes induced in the soil environment by chemical plant protection agents is highly important to maintain adequate balance of agro-ecosystems and an appropriate level of agricultural production. Monitoring of soil biological indicators is also important for environmental protection and human health. Parameters of microbiological, biochemical, and enzymatic activity have repeatedly been used for monitoring of the condition of soil environments subjected to various types of human pressure [3, 24, 40, 48]. The Biolog EcoPlate™ method is used for the assessment of the impact of different fertilizers and plant protection products as well as the presence of contaminants on soil microbiological activity [27, 31, 49]. Correlations have been demonstrated between the catabolic potential of microbial communities and other indicators of soil environment quality [39]. Changes in abundance, diversity and activity of soil microorganisms can be used to monitor soil degradation level and to improve the quality of degraded soil [2, 9, 24].

The large variety of herbicides available in the market requires relevant knowledge of their application, e.g. doses, conditions in which they can be used, and plant susceptibility to herbicides [4, 30]. Increased use of herbicides carries a risk for ecology and human health [4]. In many countries, the use of glyphosate in agricultural crops has been banned. Poland has not withdrawn glyphosate from the use following the latest scientific data and expert analyses, including the opinion of the Committee for Risk Assessment (RAC) of the European Chemical Agency (ECHA) [21]. In Poland, the use of herbicides from the group organo-phosphates which includes glyphosate amounted to 1159 tons in 2016 [11].

Poland is an important producer of rapeseed. According FAOSTAT [11] the sown acreage in 2017 was 914 thousand hectares, and yield was 29,502 hg ha^−1^. For comparison in other countries: Germany the sown acreage was 1,308,900 hectares, and yield was 32,666 hg ha^−1^; USA the sown acreage was 814 thousand hectares, and yield was 17,492 hg ha^−1^; Argentina the sown acreage was 25 thousand hectares, and yield was 19,992 hg ha^−1^.

A characteristic trait of rapeseed is its long and uneven maturation. The diversity in the level of maturity in rapeseed plantation is associated with the risk of excessive seed moisture or losses caused by mature seed shedding. To ensure uniform seed ripening and to prepare the rapeseed plantation for a one-stage harvest, a desiccation treatment is applied, i.e. plant drying via disturbances in the photosynthetic cycle.

Glyphosate herbicide is one of many chemical agents applied as desiccants available on the market. Glyphosate is a component of many herbicide formulations used for protection of agricultural and horticultural crops worldwide. Investigations conducted in Argentina by Peruzzo et al. [41] showed the presence of glyphosate in water and soil, which indicates the need to study glyphosate-containing formulations, also in terms of their impact on soil microbiological and biochemical activity [58].

The aim of the study was to analyze the response of soil microorganisms to cultivation with using glyphosate of six spring oilseed cultivars.

Literature review shows that herbicides applied in agriculture differently affect soil environment, including microorganism activity which are responsible for soil fertility and health status [35, 36, 43, 48, 57]. There is a need to conduct a comprehensive assessment of the condition of soils treated with chemical plant protection products. Due to the ambiguous research results, and no data on the impact of cultivation different oilseed cultivars the authors conducted a comprehensive study using not only traditional methods applied in soil microbiology, but also a modern method of analyzing microorganism functional diversity.

The authors hypothesized that glyphosate may affect soil microorganisms to varying degrees depending on rapeseed cultivar.

## Materials and methods

### Sampling area

The field experiments were set up in the Experimental Station for Variety Assessment in Głębokie, Kujawsko-Pomorskie Province, Poland (52º 38′ 41'' N, 18º 26′ 18'' E).

The experiment was established on soil from the class of black earths (Mollic Gleysols) formed from sandy loam, which contained 65% sand fraction (2–0.5 mm), 23% loam fraction (0.05–0.002 mm), and 12% floating particles (< 0.002 mm). The characteristics of the soil used in the experiments are provided in Table S1.

The experimental treatments (area of 12 m^2^) were established in five replications and sown spring rapeseed (*Brassica napus* L.) with using six cultivars: Belinda, Markus, Sw svinto, Tamarin, Feliks, and Clipper (Table S2). They are included in the National Register of Varieties in Poland [20]. Glyphosate was sprayed 7 days before plant harvest. The preparation was used to accelerate and even out the maturity of the rape field and reduce the losses of rape seeds caused by falling off. The technological dose recommended by the manufacturer (3 dm^3^ ha^−1^ in 100–150 dm^3^ of water) was used. Control – soil without oilseed rape and glyphosate.

### Characterisation of the Roundup formulation

A chemical Roundup 360 SL formulation was used in the field experiment. Its applicable form is a concentrate for making an aqueous solution. In its composition, Roundup 360 SL contains a biologically active compound glyphosate (a compound from the aminophosphonate group). Isopropylamine salt is the active substance contained in the herbicide at a level of 360 g/dm^3^ of the formulation. The technological dose recommended by the manufacturer (3 dm^3^ · ha^−^^1^ in 100–150 dm^3^ of water) was used.

### Soil sampling

Soil for analysis was collected to sterile containers from the 0–20 cm layer, at the following time points:

I – soon after harvest,
II – 1 month after harvest
III – 2 months after harvest

Soil samples were collected randomly from each plot. The average soil sample from each plot consisted of a mixture of five soil cores with a 3 cm diameter each. Samples were transported to the laboratory in cold conditions at 4 °C. Soil samples were sieved through a 2 mm sieve, and stored at 4 °C until analysis.

### Microbiological analysis

Determination of the number of selected groups of bacteria and fungi was performed using the plate method: total number of bacteria on the medium with soil extract [56], total number of fungi on Martin medium [32], and the number of proteolytic bacteria and fungi on the medium with gelatin [56]. The plate method consisted of a series of soil dilutions. Appropriate dilutions were poured into Petri dishes, poured over with medium and incubated at 28 °C. The colonies were counted after 3 days. Colonies with a halo around were considered in case of bacteria and proteolytic fungi.

### Biochemical analysis

The rate of the ammonification process was determined on the basis of NH_4_^+^ ion content using the Nessler method [37]. Soil samples (25 g) containing 0.1% asparagine were incubated for 7 days. Subsequently, product concentration resulting from the reaction of the Nessler reagent with NH_4_^+^ ions was determined colorimetrically in the filtrates (λ = 410 nm).

The intensity of the nitrification process was determined on the basis of NO_3_^−^ content using the brucine method [37]. Soil samples (25 g) containing NH_4_H_2_PO_4_ were incubated for 7 days. Subsequently, product concentration resulting from the reaction of brucine with NO_3_^−^ ions was determined colorimetrically in the filtrates (λ = 470 nm).

Respiratory activity was determined after the addition of 1% glucose using the titration method of Rühling and Tyler [47]. Soil sample (20 g) was incubated for 24 h with 0.2 M NaOH solution. After the incubation, BaCl_2_ was added and the excess of unbound sodium hydroxide was titrated with 0.1 HCl.

### Enzymatic analysis

Dehydrogenase activity was determined according to the Thalmann [54] method. The activity was determined in 5 g soil samples using 2,3,5-triphenyl tetrazolium chloride as a substrate, and incubating in 0.2 M tris(hydroxymethyl)aminomethane buffer (Tris–HCl pH 7,4) for 48 h, at 30 °C. Enzyme activity was determined colorimetrically (λ = 485 nm) by measuring the extinction of the produced TPF (triphenylformazan). Protease activity was determined according to the Ladd and Butler [29] method. The activity was determined in 2 g soil samples using casein as a substrate, and incubating in 0.2 M Tris -HCl buffer (pH 8,0) for 1 h at 50 °C. Enzyme activity was determined colorimetrically (λ = 700 nm), based on the amount of tyrosine produced with the Folin reagent. Urease activity was determined according to the Zantua and Bremner [59] method, in 10 g soil samples using urea solution as a substrate, and incubating for 18 h, at 37 °C. Enzyme activity was determined colorimetrically (λ = 410 nm) by measuring the extinction of the mercuric amido-oxyiodide resulting from the reaction of the Nessler reagent with NH_4_^+^ ions, produced as a result of urea hydrolysis. Acid and alkaline phosphatase activities were determined in 1 g soil samples using p-nitrophenyl phosphate disodium as a substrate, and incubating in Modified Universal Buffer (pH 6,5 and pH 11) for 1 h, at 37 °C [51]. Enzyme activity was determined colorimetrically (λ = 410 nm) by measuring the extinction of p-nitrophenol (PNP) resulting from PNPNa hydrolysis.

### Community level physiological profiling (CLPP) analysis using Biolog EcoPlates

Community level physiological profiling (CLPP) analysis showing the metabolic potential of soil microbial communities was evaluated using Biolog EcoPlates (Biolog Inc., Hayward, CA, USA). The metabolic potential of soil microbial communities was determined using 31 different carbon sources located in five groups (amines and amides, amino acids, carbohydrate, carboxylic acid, and polymers [42]. The methodology includes the following activities: 1 g of soil was weighed, transferred to conical flasks holding 99 cm^3^ of sterile 0.9% NaCl each, vortexed for 30 min at 150 rpm and at 25 °C. In the next step the samples were cooled for 30 min to 4 °C and the second step 120 mm^3^ were transferred to each of the wells in the EcoPlate and incubated in the dark at 28 °C for 144 h. The experiment was carried out three replications on each soil samples. The results were read every 24 h on the MicroStation ID system. Reduction of colourless tetrazolium chloride to red formazan (λ = 590 nm) was used as the methods to determined extent to which carbon sources were used by soil microbial communities [22]. The most intensive metabolism of carbon substrates was observed after 96–144 h of incubation. The results were expressed as Average Well-Colour Development (AWCD) and Shannon–Weaver (*H*’) indices.

### Statistical analysis

All analyses were performed in triplicate. Statistical analyzes were performed using STATISTICA.PL (10) (Stat. Soft. Inc. USA). The following analyzes were selected for the statistical evaluation: one way analysis of variance (ANOVA), PC (principal component) analysis, and the Tukey’s post-hoc HSD test at a significance level P < 0.05. The AWCD index was calculated with the formula AWCD = Σ (C-R)/95; where C = 6 absorbance in each well and R = absorbance in the control well [14]. The Shannon–Weaver (H’) index was evaluated according to Gomez et al. [16].

## Results

### Microbiological activity

**Fig. 1:**
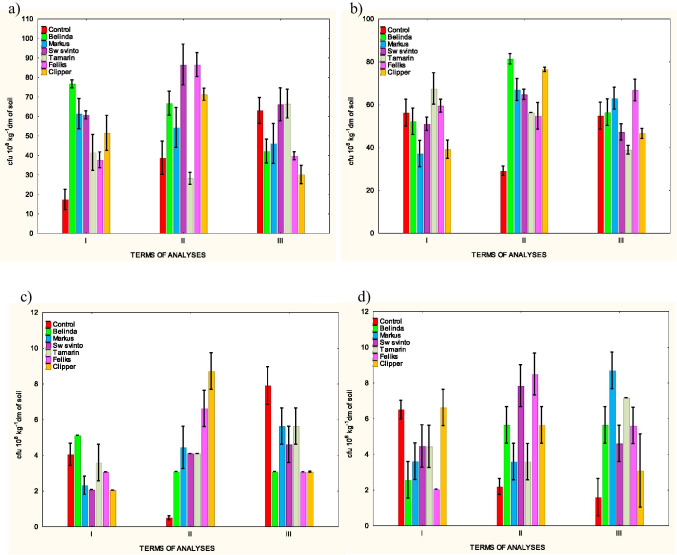
The numbers of bacteria and fungi in the soil. **a**) – total bacteria, **b**) – total fungi, **c**)—proteolytic bacteria, **d**)– proteolytic fungi

The results concerning the development of the studied groups of bacteria and fungi showed significant changes in the level of these microbiological parameters in the soil under all rapeseed cultivars after application of herbicide (Fig. 1). The intensity and direction of these changes varied depending on the time point and plant cultivar.

In the 1st and 2nd period of the study, a significant increase in the total bacteria count in the objects with plants was recorded (37.54 – 86.42 cfu 10^8^ kg^−1^) (Fig. 1a). The strongest stimulation of this group of microorganisms was found in the soil under the cultivars Belinda (76.55 cfu 10^8^ kg^−1^), Sw svinto (86.39 cfu 10^8^ kg^−1^) and Feliks (86.42 cfu 10^8^ kg^−1^). However, the number of these bacteria, recorded in time point II under the Tamarin cultivation was only at a level 20.07 cfu 10^8^ kg^−1^ which was similar to the control soil (38.58 cfu 10^8^ kg^−1^). With time, the stimulating effect disappeared, and even in some objects a decrease in the number of bacteria was noted, which was most pronounced in the soil under the cultivar Clipper (30.02 cfu 10^8^ kg^−1^).

Rapeseed cultivation with the use of the herbicide had a smaller impact on the development of filamentous fungi (Fig. 1b). Significant changes in the total number of fungi were recorded in all objects only in the second experimental period (54.56 – 81.22 cfu 10^6^ kg^−1^). There was an increase in the number of this group of microorganisms, which was most evident in the soil under the cultivar Belinda (81.22 cfu 10^6^ kg^−1^) and Clipper (76.25 cfu 10^6^ kg^−1^), and the least pronounced under Tamarin (56.14 cfu 10^6^ kg^−1^) and Feliks (54.56 cfu 10^6^ kg^−1^). There were generally no significant changes in the development of filamentous fungi at time points I and III. A decrease of this microbiological parameter was found only in the first period in the object under Markus (36.91 cfu 10^6^ kg^−1^) and Clipper (38.95 cfu 10^6^ kg^−1^) cultivation and in the third period under Tamarin cultivation (38.79 cfu 10^6^ kg^−1^).

The data presented in Fig. 1c, d shows that the cultivation of rapeseed with the use of Roundup herbicide has significantly contributed to changes in the development of proteolytic bacteria and fungi at all experimental time points. The development of protein-decomposing bacteria (Fig. 1c) was usually weaker in the soil under rapeseed in the first (2.03 – 3.04 cfu 10^8^ kg^−1^) and third (3.05 – 5.65 cfu 10^8^ kg^−1^) period, as compared to the control soil (4.03 and 7.89 cfu 10^8^ kg^−1^). This effect was evident in the object under the cultivar Clipper. In contrast to the above-mentioned time points, the development of proteolytic bacteria at time point II was stimulated in all objects (3.07 – 8.70 cfu 10^8^ kg^−1^), with the strongest effect in the soil from the cultivar Clipper. The development of protein-decomposing fungi (Fig. 1d) was clearly inhibited (2.03 – 4.45 cfu 10^6^ kg^−1^), but only in the initial period of the experiment, i.e. in the first period (exception cultivar Clipper). The strongest inhibition of this microbiological parameter was recorded in the soil under the cultivar Feliks. Over time, the negative effect weakened and starting from time point II, a stimulation of the development of these microorganisms was recorded in most objects (3.57 – 8.68 cfu 10^6^ kg^−1^). This phenomenon was the strongest in objects with the cultivars Feliks (8.47 cfu 10^6^ kg^−1^). and Markus (8.68 cfu 10^6^ kg^−1^).

### Biochemical activity

**Fig. 2:**
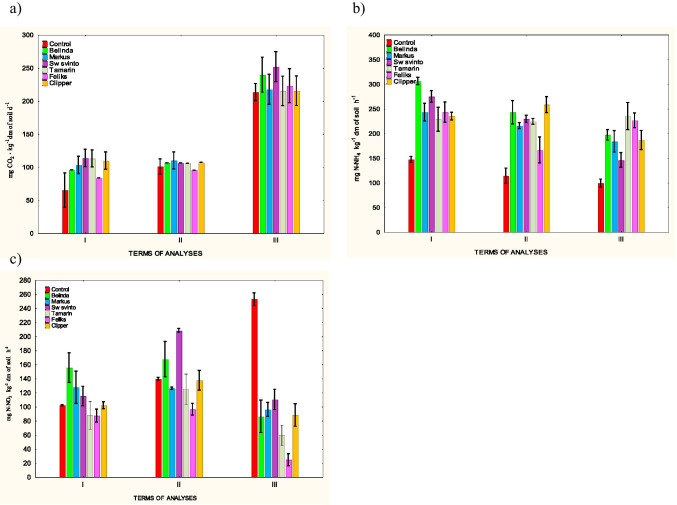
Biochemical activities in the soil. **a**) – respiration, **b**) – ammonification, **c**) – nitrification

The data presented in Fig. 2a indicate that the rapeseed cultivation with the herbicide treatment had a significant effect on the respiratory activity in the analyzed soil only in experimental time point I. Stimulation of this biochemical parameter was recorded in all treatments (83.32 – 113.56 mg CO_2_ kg^−1^). This effect was most pronounced in the treatments with Sw svinto (113.56 mg CO_2_ kg^−1^) and Tamarin (112.94 mg CO_2_ kg^−1^) cultivations and the least evident in the treatment with the Feliks cultivar (83.32 mg CO_2_ kg^−1^).

The results of the intensification of biochemical processes associated with nitrogen metabolism in soil, i.e. ammonification and nitrification (Fig. 2b, c), demonstrated that the cultivation of rapeseed treated with glyphosate herbicide significantly disturbed these processes. The effect was more pronounced in the case of the ammonification process (Fig. 2b). The cultivation induced a clear stimulation of nitrogen mineralization at all experimental treatments and time points (146.04 – 305.93 mg N-NH_4_ kg^−1^). The intensity of this process exhibited a certain dynamics of changes, and the highest values were recorded in time points I and II, especially in the Belinda cultivation treatment (305.93 and 247.73 mg N-NH_4_ kg^−1^).

In contrast to ammonification, changes in nitrification intensity were not as evident and unidirectional (Fig. 2c). At time points I and II, this process in individual treatments was inhibited, stimulated or remain unchanged. Its highest intensity was recorded at time point II in the treatment with the Sw svinto cultivation (208.26 mg N-NO_3_ kg^−1^) and the weakest intensity was detected in both time points in the objects with the cultivar Feliks (87.14 and 96.22 mg N-NO_3_ kg^−1^). The effect of the cultivation was clearly directional only at time point III. The intensity of this process was found to decline in all experimental treatments (24.53—110.14 mg N-NO_3_ kg^−1^) and was most pronounced in the soil sown with the cultivar Feliks (as in other periods).

### Enzymatic activity

**Fig. 3:**
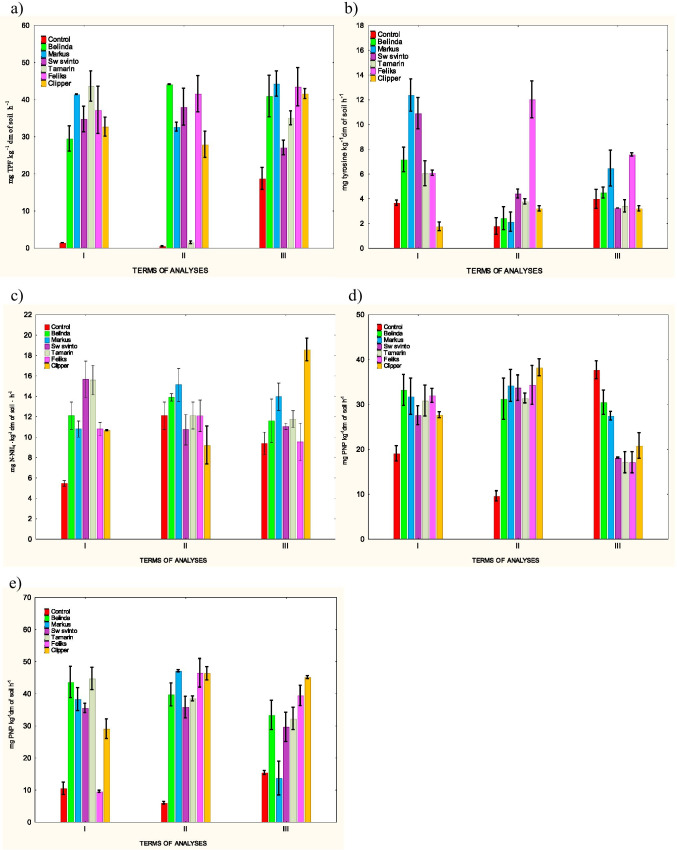
Enzymatic activities in the soil. **a**) – dehydrogenases, **b**) – protease, **c**) – urease, **d**) – acid phosphatase, **e**) – alkaline phosphatase

Similarly as for microbial abundance and biochemical processes, there were disturbances in the activity of enzymes associated with carbon, nitrogen, and phosphorus cycles in the soil with rapeseed cultivation and herbicide application (Fig. 3). The intensity and trends of these changes depended on the type of the enzyme, period, and rapeseed cultivar.

Dehydrogenase activity was clearly stimulated in all treatments and time points (1.41—44.21 mg TPF kg^−1^) (Fig. 3a). The highest values of this parameter were noted in the soil sown with the cultivars Belinda and Markus. The lowest dehydrogenase activity was detected in the soil with Tamarin cultivation; its value at time point II was similar to that recorded in the control soil (0,40 mg TPF kg^−1^).

Among the analyzed enzymes, protease and urease activities exhibited the lowest changes (Fig. 3b, c). There was a pronounced effect of the plant and herbicide on the activity of these enzymatic parameters in all treatments only in the initial period of the study (protease 6.08 – 12.35 mg tyrosine kg^−1^; urease 10.64 – 15.59 mg N-NH_4_ kg^−1^). This effect was generally positive. There was a decrease in proteolytic activity only in the treatment with Clipper cultivation (1.74 mg tyrosine kg^−1^). Over time, the positive effect declined and was noticeable only in certain treatments.

The activity of enzymes associated with phosphorus transformation, i.e. acid and alkaline phosphatases (Fig. 3d, e), showed clear changes in all treatments. The plant cultivation exerted a beneficial impact on both these parameters almost in the whole experimental period. The activity of acid phosphatase was inhibited in all treatments only at time point III (17.05 – 30.40 mg PNP kg^−1^). The beneficial effect was more pronounced in the case of alkaline phosphatase (28.99 – 47.04 mg PNP kg^−1^). The highest values of the activity of both phosphatases were noted in the soil with Clipper and Feliks cultivations.

### Biolog EcoPlates

**Fig. 4:**
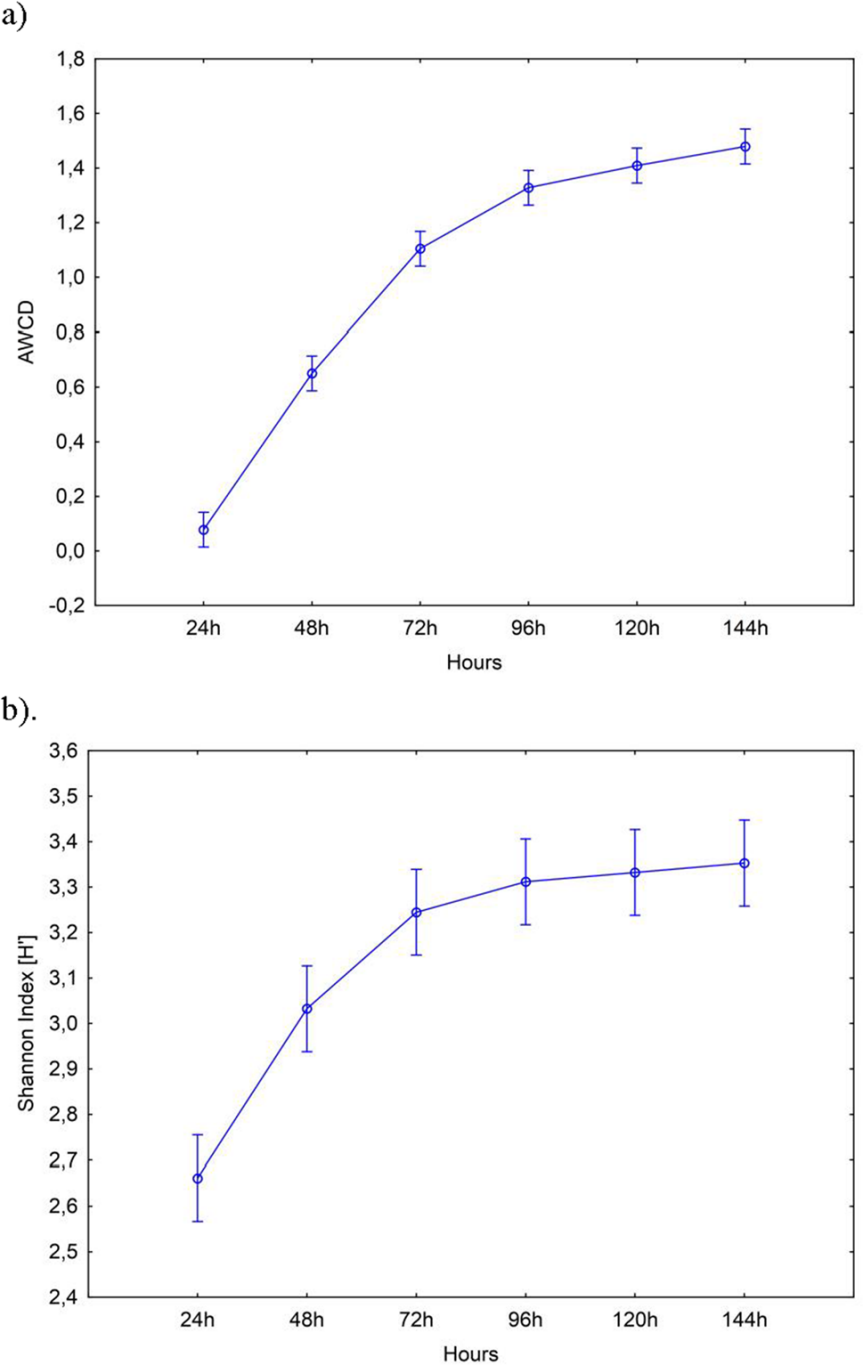
One-way analysis of variance (ANOVA) showing the changes of: **a**) AWCD index and **b**) Shannon index from time; *P* < 0,001. Vertical bars represent 95% confidence intervals

The values of the average well-color development (AWCD) index in the Biolog EcoPlate incubated from 0 to 144 h are presented in Fig. 4. The AWCD index increased proportionally to 72–144 h; therefore, 120 h were selected as the most optimal time for calculating the biodiversity index (Table S3).

There were statistically significant differences in the AWCD value in soil with different rapeseed cultivars (Table S3). The highest AWCD value was found for the soil with Belinda and Tamarin cultivations, whereas the soil sown with the cultivars Markus, Feliks, and Clipper was characterized by the lowest AWCD value. The value of the Shannon index (*H’*) also increased proportionally to the sample incubation time. The highest values of the Shannon biodiversity index were recorded at 96–144 h of sample incubation. There were no statistically significant differences in the Shannon index value in the soil with individual rapeseed cultivars during the 120-h incubation of samples (Table S3).

**Fig. 5:**
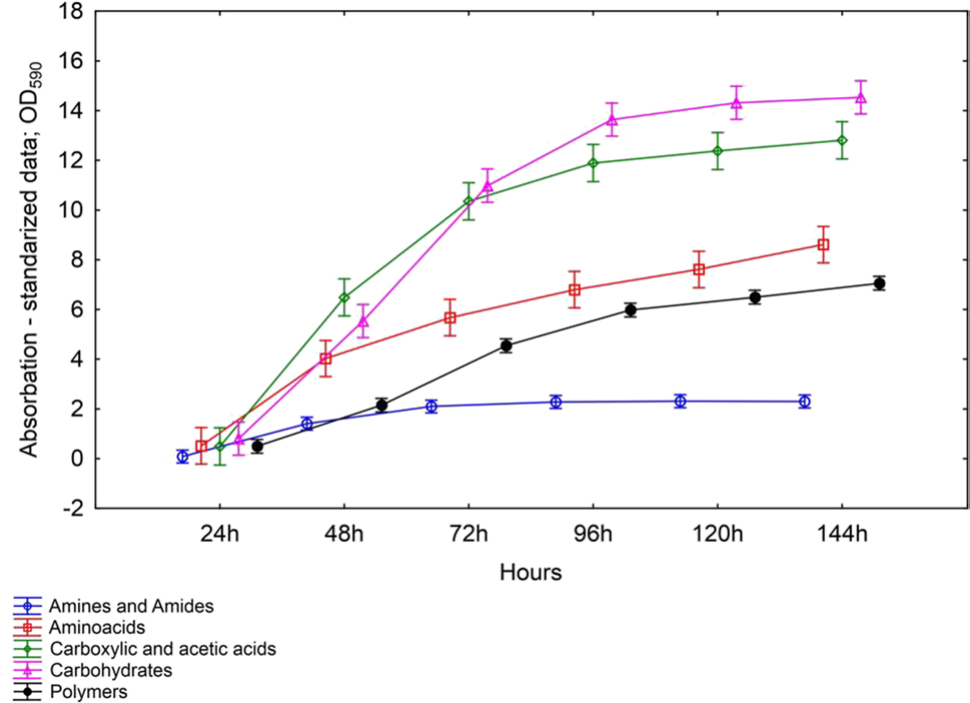
Effect of different cultivars on microbial community catabolic diversity as evaluated by substrate utilization in the Biolog EcoPlate incubated for 144 h

The effect of different plants on the catabolic diversity of microbial communities was evaluated by substrate utilization in the Biolog EcoPlate incubated for 0–144 h (Fig. 5). The utilization of the individual groups of compounds was proportional to the incubation time. The highest values of absorbance readings were recorded at 96–144 h. Carbohydrates and carboxylic acids were most easily utilized by the microorganisms. The lowest absorbance values were observed for the utilization of amines and amides as well as polymers (Fig. 5).

**Fig. 6:**
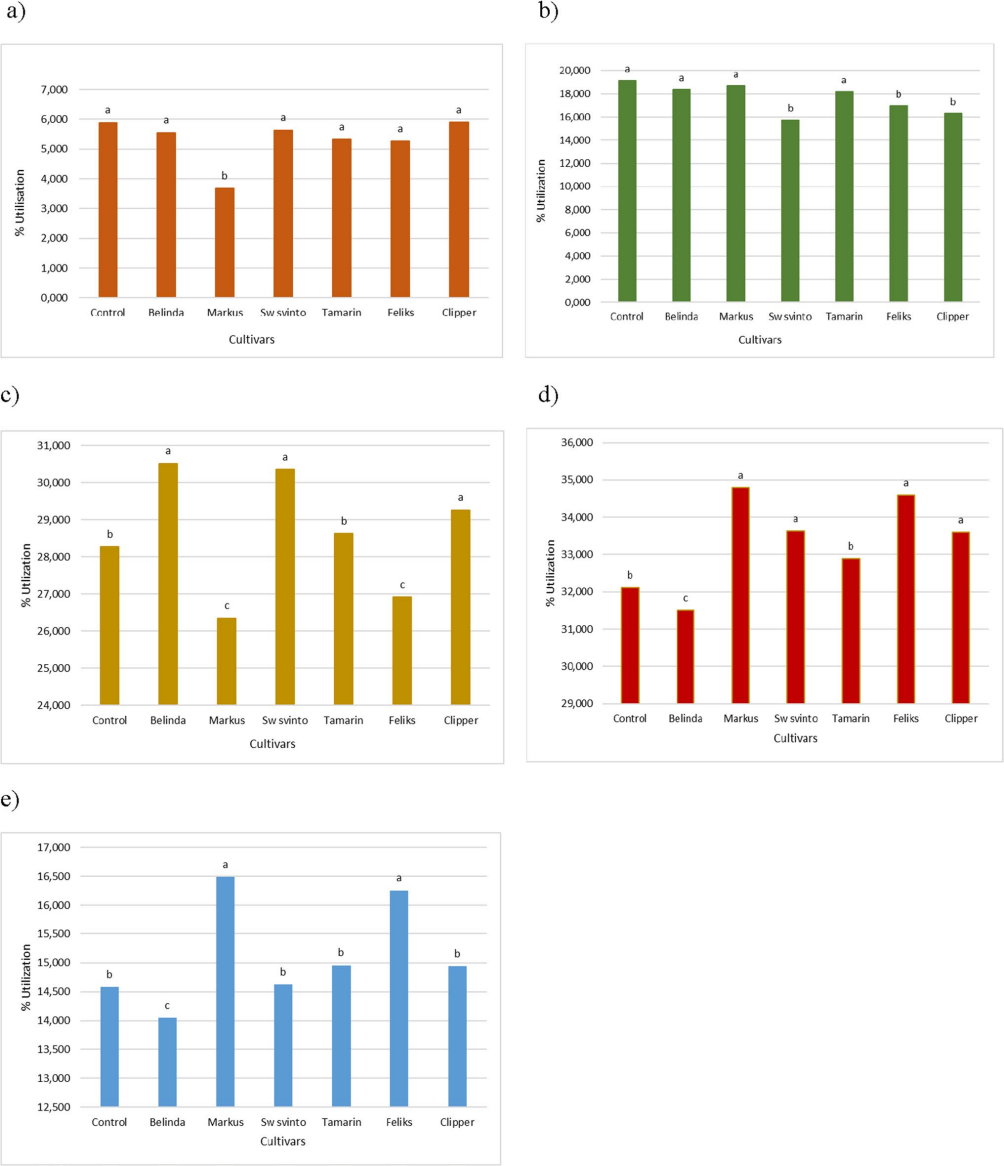
Effect of different cultivars on microbial community catabolic diversity as evaluated by substrate utilization in the Biolog EcoPlate incubated for 120 h. Vertical bars represent the standard error of the mean. Treatment means separated by different letters are significantly differ (Tukey’s mean separation test, *P* < 0.05). **a**) amines and amides, **b**) aminoacids, **c**) carboxylic and acetic acids, **d**) carbohydrates, **e**) polymers

There were statistically significant differences in the microbial utilization of individual compound groups in soils sown with different rapeseed cultivars (Fig. 6). During the 120 h incubation of Biolog EcoPlates, the highest metabolic activity of microorganisms utilizing carboxylic acids was detected in the soil with the cultivation of Belinda, Sw svinto, and Clipper. In terms of utilization of carbohydrates as a carbon source, the highest metabolic activity of microorganisms was observed in the soil sown with the cultivars Markus and Feliks. The greatest variation among the cultivars was observed in the utilization of polymers as a carbon source. In this group, the highest activity was noted for the cultivars Markus and Feliks.

**Fig. 7:**
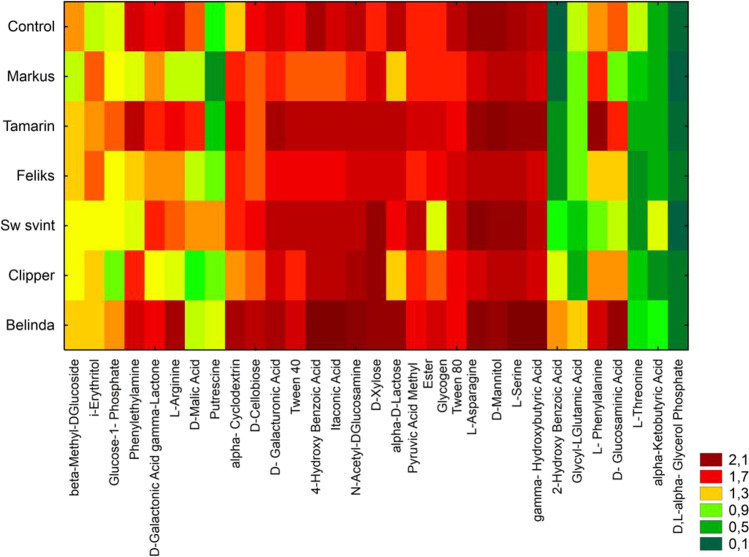
HeatMaps for the carbon utilization patterns of the substrates located only on the Biolog EcoPlates date incubated for 120 h from soil samples

Figure 7 presents a thermal map of the utilization of 31 compounds in the soil for each rapeseed cultivar during incubating the plates for 120 h. The soil collected from cultivations of the cultivars Belinda, Tamarin, and Sw svinto was characterized by the highest microbial activity.

Soil sampled from the cultivation of Belinda, Tamarin, and Sw svinto and control soil were characterized by the highest correlations between the rapeseed cultivars and the biological activity assessed on the basis of the utilization of carbon sources during the 120 h incubation of Biolog EcoPlates (Figure S1a). The principal component analysis (PCA) revealed strong correlations between soil biological activity parameters and indicators of the activity obtained using the Biolog method. There were strong positive correlations between the number of fungi and the utilization of carbohydrates and polymers by microorganisms. In turn, amino acid utilization correlated positively with the number of all and proteolytic bacteria and acid phosphatase activity (Figure S1b).

## Discussion

One of the many important roles played by microorganisms is their involvement in degradation and detoxification of various types of soil contaminants [10], e.g. chemical plant protection agents (pesticides). As shown by Upadhyay and Dutt [57], fungi, bacteria, and actinomycetes exhibit varied ability to transform and degrade pesticides. Among these microorganisms, the greatest ability to degrade xenobiotics is attributed to fungi due to their high resistance to adverse environmental conditions. Gianfreda and Rao [15] have argued that fungi mostly contribute to biotransformation of pesticides and xenobiotics in the soil by changing the structure of compounds, and thus contributing to the elimination of their toxicity. Transformed pesticides are released into the soil and are further degraded by bacteria. Therefore, soil microorganisms prevent the accumulation of these substances in the soil environment [52].

The intensity of pesticide degradation by soil microorganisms largely depends on organic matter content and climatic conditions, e.g. moisture and temperature. Humidity and temperature influence the growth of bacteria and fungi and, consequently, their activity [50]. The seasonal changes in the number of bacteria and, to a lesser extent, fungi in the control soil could be caused by fluctuations in temperature and humidity under field conditions. Stronger changes in the case of bacteria probably resulted from the greater sensitivity of these microorganisms. Stanaszek-Tomal [50] shows the high resistance of fungi to unfavorable conditions. As emphasised by many authors [7, 18, 38], an increase in soil organic matter content (crop residues), which is a source of nutrients for microorganisms, leads to an increase in microbial abundance. Sebiomo et al. [48] studied the influence of, among others, glyphosate on the populations of bacteria, actinomyces and fungi in a dose recommended by the manufacturer and found its negative impact on these microbiological parameters. In addition, organic matter contributes to herbicide immobilization in soil [12]. The present analysis of the number of soil bacteria after glyphosate formulation application in the individual experimental treatments demonstrated that the changes were dependent on rapeseed cultivar and duration of formulation treatment. This suggests a significant influence of rapeseed cultivars on these microbiological parameters.

The results of the analysis of fungal abundance in the experimental soil indicated that glyphosate significantly stimulated the growth in this microbial group in the presence of different rapeseed cultivars. Fungi, next to bacteria and actinomycetes, have the capability to use pesticides as a source of energy and C [57] Carbon-rich root secretions may have also exerted an impact on the growth of this microbial group [33]. The number of fungi increased, which may suggest that fungi are able to utilize glyphosate as an energy source. As demonstrated by Benslamaand and Boulahrouf [5], glyphosate had no negative effect on microbial activity and could improve soil quality. The authors underlined the necessity of further investigations to assess the risk of long-term application of glyphosate in various regions. Sebiomo et al. [48] investigated the effect of various herbicides, including glyphosate, on soil microbial populations. Their experiments showed that microorganisms were able to utilize herbicides as a carbon source, as evidenced by the increase in bacterial and fungal populations after glyphosate application. Partoazar et al. [40] found a significantly larger population of heterotrophic bacteria in soil contaminated with glyphosate for a longer period (12 years) than in soil treated with the formulation for 1 month or 1 day or in non-contaminated soil. Ratcliff et al. [46] demonstrated that a high glyphosate concentration stimulated microbial growth, whereas the field dose of the formulation did not induce major changes in microbial population.

A common indicator in the assessment of the soil environment status is respiratory activity, which reveals potential disturbances in carbon transformation processes induced by adverse effects of pesticides and other xenobiotics. The present field experiments did not demonstrate a negative effect on the respiratory activity of soil sown with different spring rapeseed cultivars. In the initial phase of the experiment, respiratory activity in the experimental treatments with rapeseed cultivation was higher than in the control soil. In the last period of the analyses, rapeseed cultivars did not show a significant impact on respiratory activity. Similar results were reported by other authors, e.g. Kara et al. [25].

Enzymatic activities are commonly recognized as sensitive indicators informing early about the dynamics of organic matter metabolism, circulation of nutrients, as well as about stress and restoration processes occurring in soil [8].

Soil dehydrogenases are regarded as a good indicator of total microbial activity and can be used to assess the effects of herbicide application on soil microflora [48].

Glyphosate applied in the present study significantly stimulated the activity of dehydrogenases in the soil with spring rapeseed in the initial and final periods of the experiment. The only exception was the soil with the cultivar Tamarin, in which dehydrogenase activity decreased to 1.410 mg TPF kg^−1^ day^−1^ at time point II.

Ranjith et al. [45] investigated soil dehydrogenase activity after glyphosate application in 11 different concentrations (from 0 to 2000 ppm). The authors observed higher dehydrogenase activity in the final stage of the experiment, which they explained by the ability of microorganisms to utilize less complex glyphosate-derived compounds formed after degradation carried out by specialized microbial groups. Sebiomo et al. [48] found in the study on the effect of four herbicides on dehydrogenase activity that glyphosate-treated soil exhibited the highest dehydrogenase activity in comparison to other herbicides used in the experiment. The authors concluded that this could be related to an increase in microorganism population and their ability to utilize this herbicide as a carbon source. A study carried out by Partoazar et al. [40] demonstrated that dehydrogenase activity increased substantially over time compared to control.

Protease responds to pesticides present in soil. Its activity in soil can indicate the biological ability of enzymatic transformation of the substrate. One of the most important functions of the enzyme is its involvement in mineralization of organic nitrogen compounds, which affects the availability of this element to plants [26]. The experiments with pesticides of the latter author showed an increase in soil proteolytic activity induced by low doses of these formulations. Ramudu et al. [44] found an increase in protease activity caused by the application of field doses of fungicides. Significantly higher protease activity in soils with the cultivation of spring rapeseed cultivars, especially Markus, Clipper, and Feliks, was also found in the present study.

Urease is another enzyme involved in nitrogen transformation in soil that also responds to soil contamination. As shown by Ramudu et al. [44], a decline in the activity of this enzyme in soil may be related to a decrease in ion concentration and pH as well as pesticide contamination. In the present study, urease activity was influenced by the herbicide applied and rapeseed cultivar.

Nitrogen is one of the most important biogenic elements in nature, and ammonification and nitrification are processes indicating its transformation in soil. These processes not only play an important role in the circulation of this element in soil, but are also regarded as an important indicator of soil biological activity. Muñoz-Leoz et al. [34] found that microorganisms involved in nitrogen circulation are the most sensitive soil microorganisms to soil contamination with plant protection products. In their study, Kara et al. [25] observed that triazine herbicide (active substance: 35% terbutryn, 15% terbuthylazine) caused an increase or a decrease in the intensity of the ammonification process depending on soil pH. Herbicide applied in the present study had a stimulating effect on the ammonification process, and differences in its intensity depended on the spring rapeseed cultivar. The intensity of nitrification in the field experiment exhibited significant periodic fluctuations. Rapeseed cultivars had an effect on differences in nitrification intensity. Noteworthy is the low value of this indicator at time point II of the analyses in the soil sampled from all treatments with the cultivation of rapeseed cultivars. In this period, the intensity of ammonification in the soil sown with the analyzed rapeseed cultivars was significantly higher than in the control soil.

The activity of enzymes involved in phosphorus transformation in soil can also be used to assess the impact of pesticides on changes in the soil environment. The current study demonstrated that acid and alkaline phosphatase activity at time points I and II was significantly higher in the soil from treatments with rapeseed cultivars than in control. In the final period of research, there was a decline in acid phosphatase activity in treatments with six spring rapeseed cultivars in comparison to the control soil (Fig. 3). Alkaline phosphatase activity during this study period was statistically significantly higher than in control.

The analysis of catabolism of carbon sources by soil microorganisms revealed the highest level of utilization of substrates from the carbohydrate group by all rapeseed cultivars and the lowest level in the group of amines and amides (Figure S1). Similar results on the utilization of amines and amides as well as carbohydrates by soil microorganisms were reported by other researchers [13, 53].

Literature data [23] demonstrate that cultivated plants have a greater impact on the diversity of microbial communities than soil type. Similarly, Cardozo Junior et al. [9] showed that catabolic activity was dependent on the plant cover. The results obtained in the present experiment supported this theory, as there were statistically significant differences in the AWCD values (Table S2) and in the level of substrate group utilization (Figure S1) between rapeseed cultivars.

We found correlations between different soil quality parameters and microorganism catabolic potential, which is consistent with literature data [1].

## Conclusions

The rapeseed cultivation using herbicide showed significant changes in the growth of the analyzed groups of bacteria and fungi and their biochemical and enzymatic activities in the soil. The nature and intensity of the impact on activity parameters depended on the period and plant cultivar. It should be noted that the cultivar Clipper turned out to be the least favorable plant for most of the analyzed microbiological activities.

Respiratory activity and the ammonification process were stimulated in treatments with rapeseed cultivars treated with the herbicide. The cultivar Sw svinto had a positive effect on the intensity of respiration and nitrification, whereas the cultivar Feliks exerted the least beneficial impact on these parameters. The highest increase in the ammonification process was recorded for soil from the cultivation of the cultivar Belinda.

There was a generally positive effect of rapeseed cultivars treated with the herbicide on enzymatic activity. In the case of dehydrogenase activity, this effect persisted throughout the research period and was particularly pronounced in Belinda and Markus cultivation treatments. The activity of protease and urease underwent the lowest changes. Phosphatase activity was intensified in the soil sampled from treatments with rapeseed cultivars. The cultivars Clipper and Feliks were found to exert the most beneficial effects on the activity of both acid and alkaline phosphatases.

The cultivars Belinda, Tamarin, and Sw svinto exerted the most beneficial effect on the catabolism of soil microorganisms. Simultaneously, soil originating from the treatment with the Belinda cultivation exhibited the greatest microbial diversity, as evidenced by the Shannon index (*H’*). The cultivar Markus was characterized by the least favorable effect on microbial metabolism.

The decrease in microbial activity is one of the causes of soil degradation. Cultivation of specific plant varieties that reduce the negative effect of herbicides used in agriculture may be one of the methods to prevent soil degradation. In our research, Belinda turned out to be a cultivar, under the cultivation of which an increase in the activity of microorganisms was recorded most frequently compared to soil not sown with rapeseed.

## Supplementary Information

Below is the link to the electronic supplementary material.

Supplementary file1 (DOCX 113 KB)
