# Supplementary material for: Microbial activity and community level physiological profiles (CLPP) of soil under the cultivation of spring rape with the Roundup 360 SL herbicide
Source: J Environ Health Sci Eng. 2021 Nov 12;19(2):2013–26. doi: 10.1007/s40201-021-00753-3 (PMC8638805; doi:10.1007/s40201-021-00753-3)
Supplement: Supplementary file 1 — Supplementary file1 (DOCX 113 KB) [file 40201_2021_753_MOESM1_ESM.docx]

**TABLE S1** Chemical characteristics of soil used in the experiment

| **Parameter** | **Unit** | **Value** |
| --- | --- | --- |
| Reaction | pH_KCl_ | 5.9 |
| C_org_ | g kg^-1^ d.m. | 9.7 |
| Total N | g kg^-1^ d.m. | 1.3 |
| C:N |  | 7.5 |
| Total P | g kg^-1^ d.m. | 0.7 |
| K | g kg^-1^ d.m. | 0.1 |
| Zn | mg kg^-1^d.m. | 32.5 |
| Cd | mg kg^-1^d.m. | 0.14 |
| Cu | mg kg^-1^ d.m. | 11.0 |
| Pb | mg kg^-1^ d.m. | 9.2 |
| Ni | mg kg^-1^ d.m. | 7.3 |
| Cr | mg kg^-1^ d.m. | 13.9 |
| Hg | mg kg^-1^ d.m. | 0.1 |
| Hh | cmol kg^-1^d.m. | 1.38 |

**TABLE S2** Seed characterization of individual rapeseed cultivars (<https://agro-technika.pl/archiwa/rzepak-jary-odmiany-i-uprawa/>).

| **Cultivar** | **Belinda** | **Markus** | **Sw svinto** | **Tamarin** | **Feliks** | **Clipper** |
| --- | --- | --- | --- | --- | --- | --- |
| **Seed yield (t ha^-1^)** | 10.4 | 10.5 | 9.6 | 10.3 | 9.5 | 9.8 |
| **Fat content**  **(% d.m.)** | 44.4 | 44.6 | 44.9 | 43.3 | 44.1 | 46.7 |

**TABLE S3.** Effect of different plant cultivars on the catabolic diversity of microbial community as evaluated by Shannon’s diversity index (*H*) and average well-color development (AWCD_590_) in the Biolog EcoPlate incubated for 120 h. The values are means ± standard error (*n*=3). Treatment means marked with different letters are significantly different (Tukey’s mean separation test, *P* < 0.05)

| Combinations | *H’* | AWCD_590_ |
| --- | --- | --- |
| Control | 3.340 ± 0.011 | 1.433^c^ ± 0.070 |
| Belinda | 3.352 ± 0.018 | 1.646^d^ ± 0.183 |
| Markus | 3.303 ± 0.046 | 1.220^ab^ ± 0.208 |
| Sw svinto | 3.325 ± 0.017 | 1.413^c^ ± 0.191 |
| Tamarin | 3.332 ± 0.022 | 1.503^d^ ± 0.086 |
| Feliks | 3.336 ± 0.028 | 1.335^b^ ± 0.067 |
| Clipper | 3.336 ± 0.006 | 1.312^b^ ± 0.145 |

a)


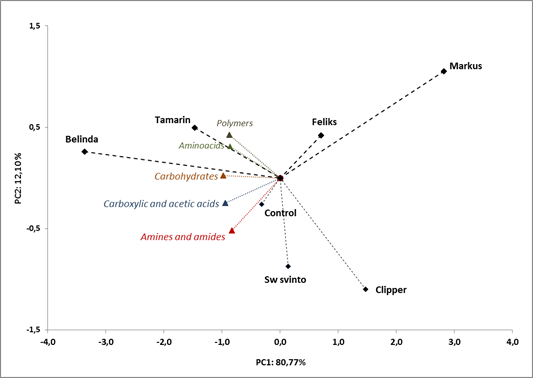


b)


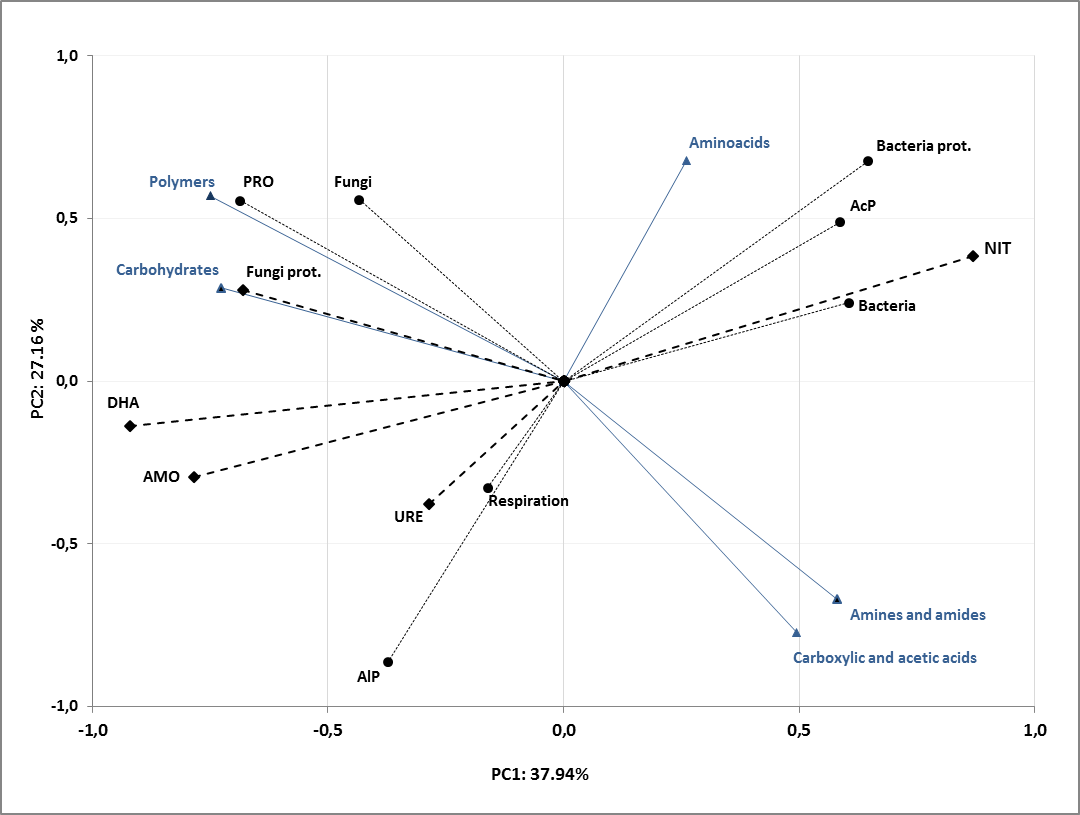


**Fig. S1**

**Fig. S1** Principal component analysis (PCA) of Biolog EcoPlates date incubated for 120h from soil samples. a) bi-plot analysis; the main groups of substrate utilization in the Biolog EcoPlate and plants; b) the main groups of substrate utilization in the Biolog EcoPlate and parameters of soil biological activity
